# Bone Marrow-Derived Mesenchymal Stem Cells Repaired but Did Not Prevent Gentamicin-Induced Acute Kidney Injury through Paracrine Effects in Rats

**DOI:** 10.1371/journal.pone.0044092

**Published:** 2012-09-06

**Authors:** Luciana A. Reis, Fernanda T. Borges, Manuel J. Simões, Andrea A. Borges, Rita Sinigaglia-Coimbra, Nestor Schor

**Affiliations:** 1 Nephrology Division, Department of Medicine, UNIFESP/EPM, São Paulo, Brazil; 2 Histology and Biology Structural Division, Morphology Department, UNIFESP/EPM, São Paulo, Brazil; 3 Electronic Microscopy Center, UNIFESP/EPM, São Paulo, Brazil; University of Torino, Italy

## Abstract

This study evaluated the effects of bone marrow-derived mesenchymal stem cells (BMSCs) or their conditioned medium (CM) on the repair and prevention of Acute Kidney Injury (AKI) induced by gentamicin (G). Animals received daily injections of G up to 20 days. On the 10^th^ day, injections of BMSCs, CM, CM+trypsin, CM+RNase or exosome-like microvesicles extracted from the CM were administered. In the prevention groups, the animals received the BMSCs 24 h before or on the 5^th^ day of G treatment. Creatinine (Cr), urea (U), FENa and cytokines were quantified. The kidneys were evaluated using hematoxylin/eosin staining and immunohystochemistry. The levels of Cr, U and FENa increased during all the periods of G treatment. The BMSC transplantation, its CM or exosome injections inhibited the increase in Cr, U, FENa, necrosis, apoptosis and also increased cell proliferation. The pro-inflammatory cytokines decreased while the anti-inflammatory cytokines increased compared to G. When the CM or its exosomes were incubated with RNase (but not trypsin), these effects were blunted. The Y chromosome was not observed in the 24-h prevention group, but it persisted in the kidney for all of the periods analyzed, suggesting that the injury is necessary for the docking and maintenance of BMSCs in the kidney. In conclusion, the BMSCs and CM minimized the G-induced renal damage through paracrine effects, most likely through the RNA carried by the exosome-like microvesicles. The use of the CM from BMSCs can be a potential therapeutic tool for this type of nephrotoxicity, allowing for the avoidance of cell transplantations.

## Introduction

Acute kidney injury (AKI) is a syndrome of rapidly declining renal function induced by a number of different insults [1].

The mortality rate of hospital acquired AKI currently ranges from 30 to 80%, and recent dialysis techniques, such as continuous renal replacement therapy and others, have had no significant impact on overall mortality. Furthermore, the efforts to develop new pharmacological therapies have been largely unsuccessful [2].

Gentamicin (G) is an aminoglycoside antibiotic widely used for the treatment of gram-negative infections [1], and it is a well-known cause of renal toxicity mainly characterized by proximal tubular necrosis, alterations in glomerular hemodynamics, and a decrease in both the glomerular plasma flow and in the ultrafiltration coefficient [3]–[6]. The use of toxic antibiotics is increasing due to antimicrobial resistance, especially in intensive care units; therefore, new strategies are necessary to treat or prevent G-induced nephrotoxicity.

BMSCs, also known as marrow stromal cells [7] or mesenchymal progenitor cells [8], are defined as self-renewable, multipotent progenitor cells with the capacity to differentiate into several distinct mesenchymal lineages [9]–[14]. Several studies have suggested that stem cells may protect AKI experimental models from cisplatin, glycerol and ischemia-reperfusion injury [10]–[12], but the mechanism by which BMSCs functionally contribute to renal tubule regeneration in AKI is a matter of debate. These experimental models are characterized by extensive necrosis of the proximal and distal tubules that may favor BMSCs migration into these areas. However, the therapeutic potential of exogenously administered BMSCs in a model of G-induced AKI has not been evaluated.

The mechanism for the effects of BMSCs remains controversial because some studies have reported that the injected BMSCs infiltrate the kidney and directly populate the injured renal tubule [12]. Other studies have found only transient evidence of injected BMSCs in the renal vasculature and no evidence for direct BMSCs incorporation into the tubules during the repair processes [13], suggesting paracrine effects.

This study was undertaken to evaluate if BMSCs were capable of minimizing or preventing the AKI induced by G. In our experiments, the cells were localized in the injured kidney, reducing the nephrotoxic effects of G. Interesting, the BMSC conditioned medium (CM) also minimized the G effects, supporting the hypothesis that BMSCs may exert a paracrine effect on the filtration function and in the damaged tissue. Because the protective effect of the CM was blunted by RNase during a long period of incubation, these paracrine effects may be mediated mainly by the RNA-like factors released by BMSCs into the medium. Therefore, in future clinical applications, it may be possible to treat this nephrotoxicity using CM alone, thus avoiding cell transplantations.

## Results

**Figure 1:**
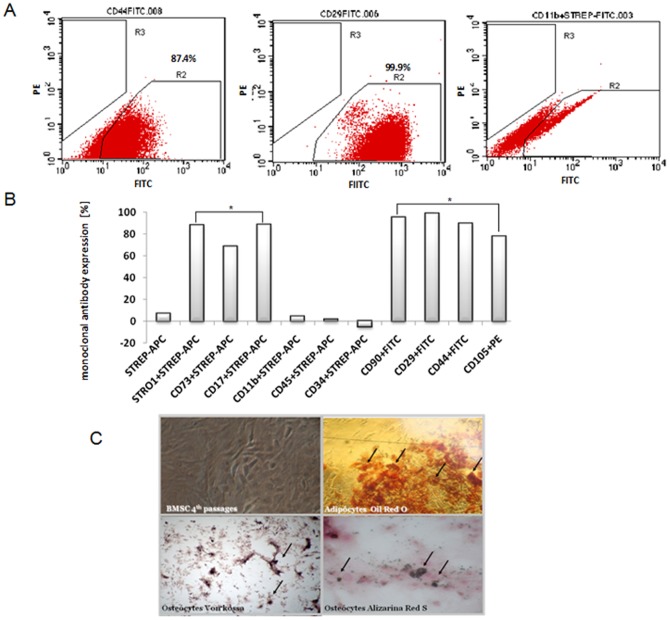
(A) FACS analysis and (B) quantification showing the monoclonal antibody expression [%] of BMSCs. Data are expressed as the mean ± S.E.M. (* p<0.05) vs. CD45, CD34 and CD11b. (C) The differentiation of BMSCs into adipocytes and osteocytes. Undifferentiated (non-induced) BMSCs were maintained in the control medium as a negative control.

First, we characterized the BMSCs by flow cytometry and the analysis of the cell differentiation. Figure 1A and B show the flow cytometry of the BMSCs cultured on the 4^th^ passage. There was a positive staining in BMSCs for STRO1 (88.1±3.8%), CD73 (68.5±17.7%), CD17 (88.6±4.1%), CD90 (95±3.6%), CD29 (98.8±1.0%), CD44 (90±1.7%) and CD105 (78±13.0%) when compared to the negative control. There was no positive staining for CD11b (4.4±0.5%), CD45 (1.5±0.4%) and CD34 (1.8±0.8%), and all of them were used as negative controls for BMSCs. Figure 1C also shows the differentiation of BMSCs into adipocytes and osteocytes.

**Figure 2:**
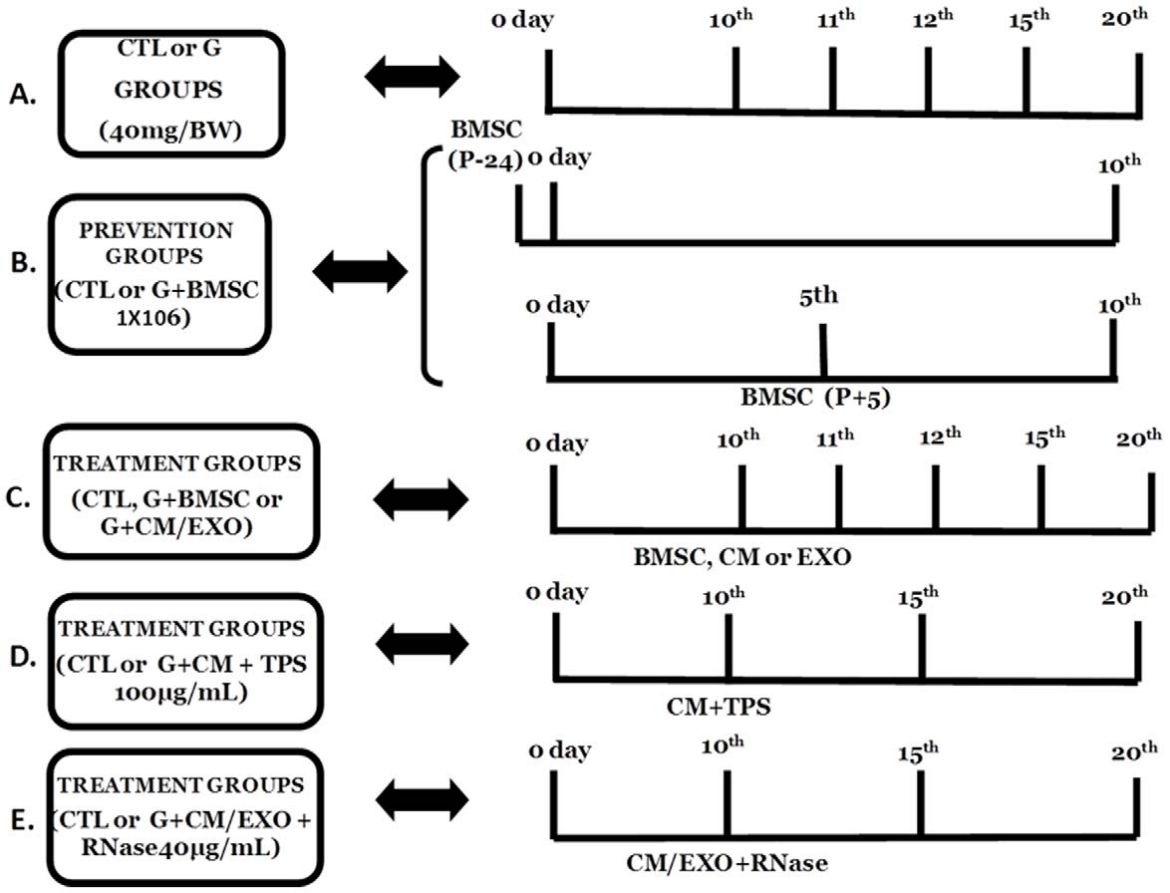
The schematic of the experiment protocol. In the control group (CTL), the rats were treated with daily i.p. injections of a vehicle (water), while the G groups received gentamicin (40 mg/Kg BW) continuously for 10, 11, 12, 15 or 20 days. For the prevention groups, the rats received BMSC 1×106 i.v. injections 24 hours before (G_10_+BMSC_−1_) or in the 5th day (G10d+BMSC5) of a 10-day treatment with G. For the treated groups, the rats received BMSC 1×106 i.v. injections on the 10th day of G treatment and continued receiving G for an additional 1 (G_11_+BMSC_10_), 2 (G_12_+BMSC_10_), 5 (G_15_+BMSC_10_) or 10 (G_20_+BMSC_10_) days. For the conditioned media protocol, the rats received G (40 mg/Kg/BW, i.p., daily) or water (CTL) for 15 or 20 days, and on the 10th day of the G treatment, the animals received 500 µl of a single dose of CM. In some experiments, the BMSCs were cultured for 12 h with trypsin and a concentration of 100 ug/ml (CM+TPS, 100 µg/ml) or RNase A at a concentration of 40 ug/ml, 280 Units (CM+RNAse) in DMEM without FBS.

To analyze the hypothesis that BMSCs could treat the AKI induced by G, the cells were transplanted into the AKI rats. Table 1 presents the serum creatinine (Cr), urea and FENa levels in the Wistar rats treated with a vehicle (CTL) or G for 10, 11, 12, 15 or 20 days and the corresponding G groups treated with BMSCs (Figure 2A and C).

**Table 1:** The biochemical results from the treatment groups transplanted with BMSCs.

GROUPS	sCr (mg/dl)	sU (mg/dl)	FENa (%)
CTL_10d_	0.5±0.02	24±2.0	0.6±0.02
CTL_11d_	0.6±0.03	26±3.0	0.7±0.02
CTL_12d_	0.7±0.02	32±6.0	0.8±0.05
CTL_15d_	0.7±0.04	29±4.0	0.8±0.05
CTL_20d_	0.5±0.03	34±8.0	0.6±0.02
G_10d_	1.9±0.07*	98±2.0*	1.5±0.04*
G_11d_	2.3±0.04*	104±1.0*	1.7±0.03*
G_12d_	2.7±0.03*	119±1.0*	1.7±0.01*
G_15d_	2.9±0.04*	128±5.0*	2.1±0.04*
G_20d_	3.4±0.08*	104±1.0*	1.5±0.04*
G_11_+BMSC_10_	1.0±0.04#	64±2.0#	1.0±0.02#
G_12_+BMSC_10_	0.7±0.03#	54±1.0#	0.7±0.01#
G_15_+BMSC_10_	0.6±0.01#	41±1.0#	0.7±0.03#
G_20_+BMSC_10_	0.9±0.02#	68±3.0#	1.0±0.01#

Data are expressed as the mean ± S.E.M. One-Way ANOVA; (p<0.05).

* *vs*. CTL_11,12,15 or 20d_; *p<.05*.

# *vs.G_11,12,15_ or _20d_*.

Serum Cr, U concentrations and FENa after 10, 11, 12, 15 and 20 days of G treatment increased in comparison to the CTL group (p<0.05) (Table 1). When the animals were transplanted with a single injection of BMSCs on the 10^th^ day, there was a significant decrease in the serum Cr, U and FENa in comparison to the G only group at all days of the treatment with the antibiotic (Table 1). This result suggests that one single injection of BMSCs could inhibit the AKI induced by up to 20 days of G administration.

Because it was observed that BMSC transplantation could inhibit the AKI induced by G, we determined whether these cells could prevent this injury when transplanted before the G treatment or before significant alteration in the functional parameters of Cr, U and FENa.

Table 2 shows the biochemical parameters in the prevention groups, where the animals were transplanted with BMSC on the 1^st^ day before the beginning of the G treatment or at the 5^th^ day of the treatment with G; no significant differences were observed in the functional parameters (Figure 2A and B). It was observed that there were no differences in Cr, U and FENa between the G group and the G group transplanted with BMSCs one day before the treatment with this antibiotic. Additionally, the BMSC transplantation after 5 days of treatment with G did not inhibit the increase in functional parameters induced by the antibiotic after 10 days of treatment (Table 2).

**Table 2:** The biochemical results for the prevention groups transplanted with BMSCs.

GROUPS	sCr (mg/dl)	sU (mg/dl)	FENa (%)
CTL_10d_	0.5±0.02	24±2.0	0.6±0.02
G_10d_	1.9±0.07*	98±2.0*	1.5±0.04*
G_10d_+BMSC_−1_	1.2±0.04*	73±1.0*	1.2±0.01*
G_10_+BMSC_5_	1.8±0.03*	87±1.0*	2.0±0.03*

Data are expressed as the mean ± S.E.M. One-Way ANOVA; (p<0.05).

* *vs*. CTL_10d_.

**Figure 3:**
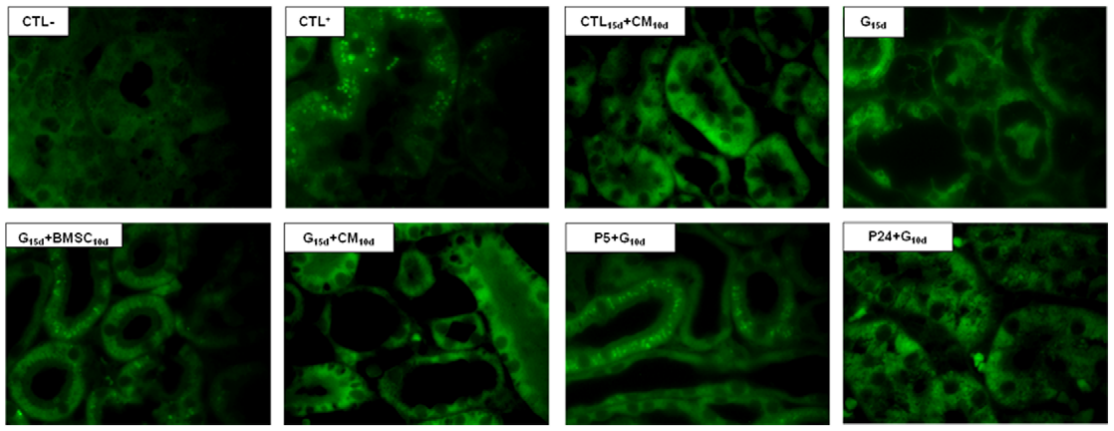
The light micrographs of the kidney sections were stained with immunofluorescence for Y chromosome (100×) for the following groups: the Male Wistar rats that received G vehicle (CTL+), the Female Wistar rats that received G vehicle (CTL−), the Female Wistar rats that received G vehicle and CM on the 10^th^ day (CTL_15_+CM_10_), the Female Wistar rats treated with G for 15 days (G_15_), the Female Wistar rats treated with G for 15 days and with male BMSCs on the 10th day (G_15_+BMSC_10_) and the Female Wistar rats treated with G for 15 days and MC on the 10th day (G_15_+MC_10_). The arrows show the presence of the Y chromosome in the epithelial tubule cells. The images are magnified 100×.

It is reasonable to suggest that the protective effect of the BMSCs was not observed because there was no homing of the BMSCs in the prevention group. Therefore, we analyzed the presence of the Y chromosome of BMSCs from male rats in the kidney of the female animals treated with G (Figure 3). All of the groups treated with G were analyzed, but in the next sections, we show the results from the prevention groups and from the animals treated with G for 15 days that received BMSC on the 10^th^ day because the injury was more severe in this group. Additional information can be found in Figure S1.

The Y chromosome was detected in the interstitial and peritubular area of the kidney in male rats (used as the positive control) and in female rats treated with G for 15 days that received BMSCs after 10 days of treatment (G_15_+BMSC_10_). Nevertheless, we did not localize the Y chromosome in the kidney of the rats treated with G for 10 days when the cells were transplanted 24 h before the beginning of the antibiotic administration (P-24+G_10_). This result corroborates the hypothesis that the injury stimulus is necessary for the homing of the cells. Interestingly, we detected the Y chromosome in the kidney of the animals treated with G for 10 days that received the BMSC at the 5^th^ day (Figure 3).

It has been shown that the main mechanism of action of BMSCs is paracrine [31]. To further study this mechanism, we analyzed the hypothesis that the conditioned medium (CM) of BMSCs could also inhibit the G-induced AKI. Table 3 shows the effects of a single injection of the CM of BMSCs on the G-induced AKI. The Cr, U and FENa levels decreased in the G group treated with CM on the 10^th^ day of the antibiotic treatment in comparison to the group treated with G alone for 15 days (p<0.05).

**Table 3:** The biochemical results for the treatment groups transplanted with CM.

GROUPS	sCr (mg/dl)	sU (mg/dl)	FENa (%)
CTL_15d_	0.7±0.04	29±4.0	0.3±0.01
G_15d_	2.7±0.03*	119±1.0*	1.7±0.03*
G_15_+CM_10_	0.6±0.01#	41±1.0* ^,^ #	0.7±0.03#
G_15_+RNAse_10_	3.4±0.08*	128±5.1*	2.2±0.03*
G_15_+CM+RNAse_10_	3.1±0.03*	106±4.9*	1.4±0.03*
G_15_+TPS_10_	2.1±0.09*	102±3.3*	1.9±0.04*
G_15_+CM+TPS_10_	1.0±0.01+	99±2.7* ^,^ +	1.2±0.02* ^,^ +

**Data are expressed as the mean ± S.E.M. One-Way ANOVA; (p<0,05).**

* ***vs. CTL; p<.05.***

# ***vs.G_15d_; p<.05.***

+ ***vs.G_15_+TPS_10_.***

**Figure 4:**
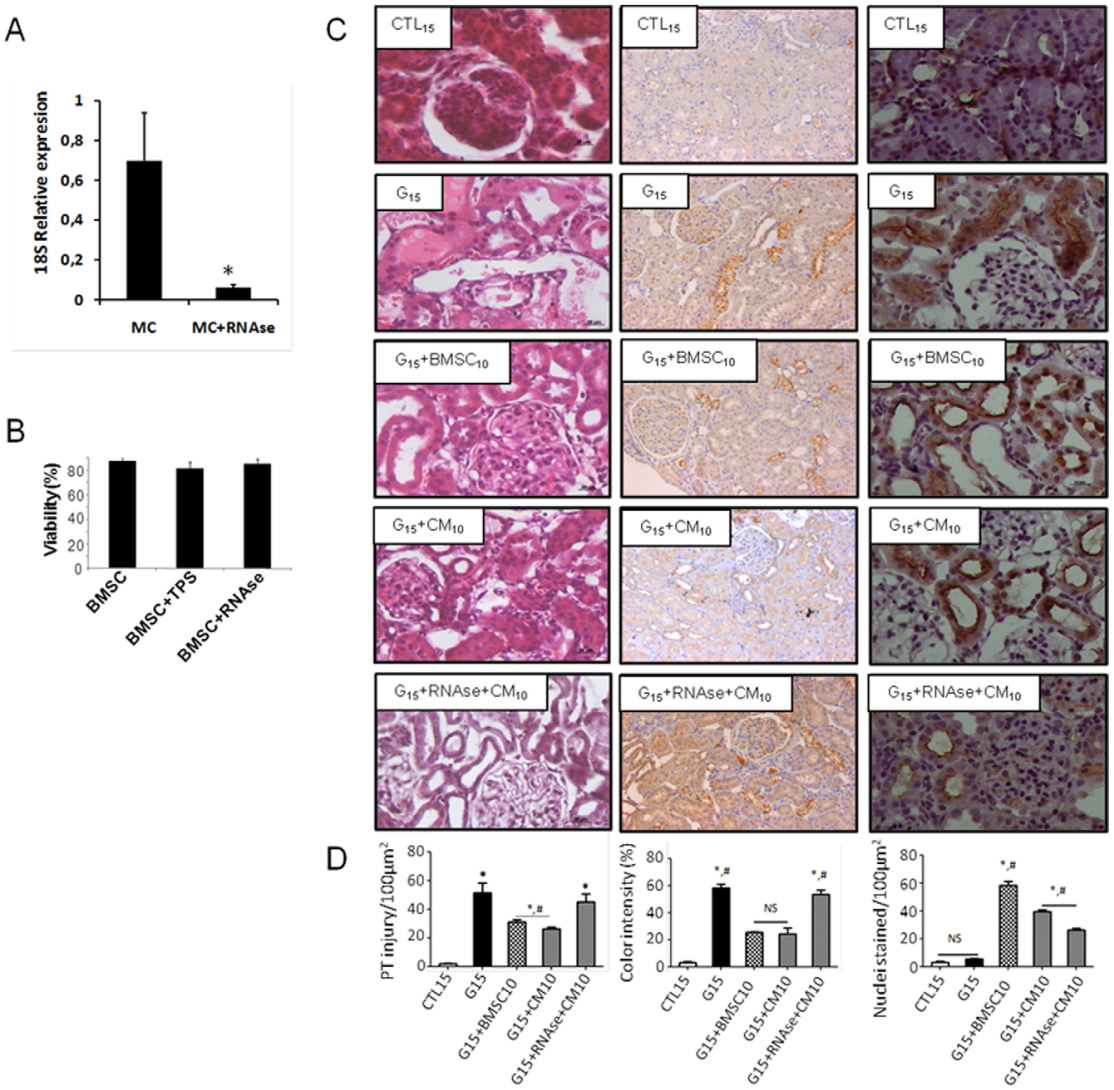
(A) RT-PCR for 18S RNA in culture medium (CM) from BMSC controls and culture medium treated with RNase (CM+RNAse). (B) BMSC viability after treatment with Trypsin and RNase by Trypan blue extrusion. (C) The light micrographs of the kidney sections stained with hematoxylin eosin and with immunochemistry for caspase 3 and KI67. (D) A graphic representation showing the quantitative analysis of the control rat micrographs (CTL_15_; CTL_15_+BMSC_10_). The histology of the kidney tissue from rats treated with G for 15 days (G_15_). Some groups presented with massive tubular necrosis and unstained nuclei (G_15_, G_15_+RNAse+CM_15_), while other groups presented with intensely stained nuclei (G_15_+BMSC_10_, G_15_+CM_10_). Data are expressed as the mean ± S.E.M. (* p<0.05) vs. CTL15d, (# p<0.05) vs. G15.

Additionally, several studies have suggested that the paracrine effect of BMSCs is mediated by protein or RNA (microRNA or mRNA carried by microvesicles) [21]. To address this question, we treated the conditioned medium with RNase A and trypsin for 12 h prior to injecting it into the rats. The protective effect of CM was blunted after it was treated with RNase for 12 h (Table 3). Figure 4A shows the 18S housekeeping gene expression in the conditioned medium. The treatment with RNase (40 µg/ml) significantly degraded the RNA in conditioned medium samples. When the CM of BMSC was incubated with trypsin and was administered to the rats during G treatment, the protective effect of CM was not completely blunted, suggesting that the soluble proteins in the CM are not entirely responsible for the protective effect; another protective mechanism may contribute to this effect (Table 3).

It is possible that the treatment of BMSCs with RNase A or trypsin could damage the cells; to address this question, we analyzed the viability of BMSCs either untreated or treated with trypsin or RNase for 12 hours. However, there was no significant difference in cell viability between BMSCs that were untreated or treated (Figure 4B).

To evaluate the impact of the transplantation of BMSCs or its CM on the severity of the tissue damage induced by G in the kidney, a histological scoring was performed based on the number of proximal tubules with damage (Figure 4C).

There was a significant increase in the histological scoring in the G_15_ (51.6±2.3 injuries/100 µm^2^) treated group compared with the CTL group (2±0.06 injuries/100 µm^2^) (p<0.05). The BMSCs transplanted or the CM injected on the 10^th^ day significantly protected the kidney from G administration over 15 days, as observed in groups G_15_+BMSC_10_ (35.8±4.9 injuries/100 µm^2^) and G_15_+CM_10_ (31±1.9 injuries/100 µm^2^) (p<0.05). However, when the animals were treated with the CM exposed to RNase (G_15_+CM_10_+RNAse_10_), the lesions were maintained (43.6±3.4 injuries/100 µm^2^). It is interesting that in the prevention groups, the effects of G were minimized when the BMSCs were given on the 5^th^ day of the treatment, but this result was not corroborated by the functional parameters. The results for the other G-treated groups can be found in Figure S2.

Figure 4C shows the immunohistochemistry for apoptotic cell death and proliferation, as shown by its regulatory protein caspase 3 and the nuclear protein KI67. There was an increase in caspase 3 staining in the G_15_ (58.2±2.8%) group versus the G_15_+BMSC_10_ (24.4±4.2%) or G_15_+CM_10_ (25.5±0.5%) groups (p<0.05). The protective effect of the CM was blunted when it was incubated with RNase.

For the proliferation of the cells, the G_15_+BMSC_10_ (55.5±0.5 nuclei/100 µm^2^) and G_15_+CM_10_ (58.2±2.8 nuclei/100 µm^2^) animals showed an increase in the nuclear expression of KI67 in comparison to the G_15_ (5.2±0.4 nuclei/100 µm^2^; p<0.05) and CTL_15_ (3.2±0.4 nuclei/100 µm^2^) (p<0.05) animals. The proliferation induced by the CM was inhibited when the CM was treated with RNase (39.3±1.1 nuclei/100 µm^2^) (p<0.05). The animals treated with only BMSC or CM showed no increase in KI67 (Figure 4D).

To further investigate the mechanism of protection in the CM, we extracted exosome-like microvesicles from CM treated or not with RNase.

**Figure 5:**
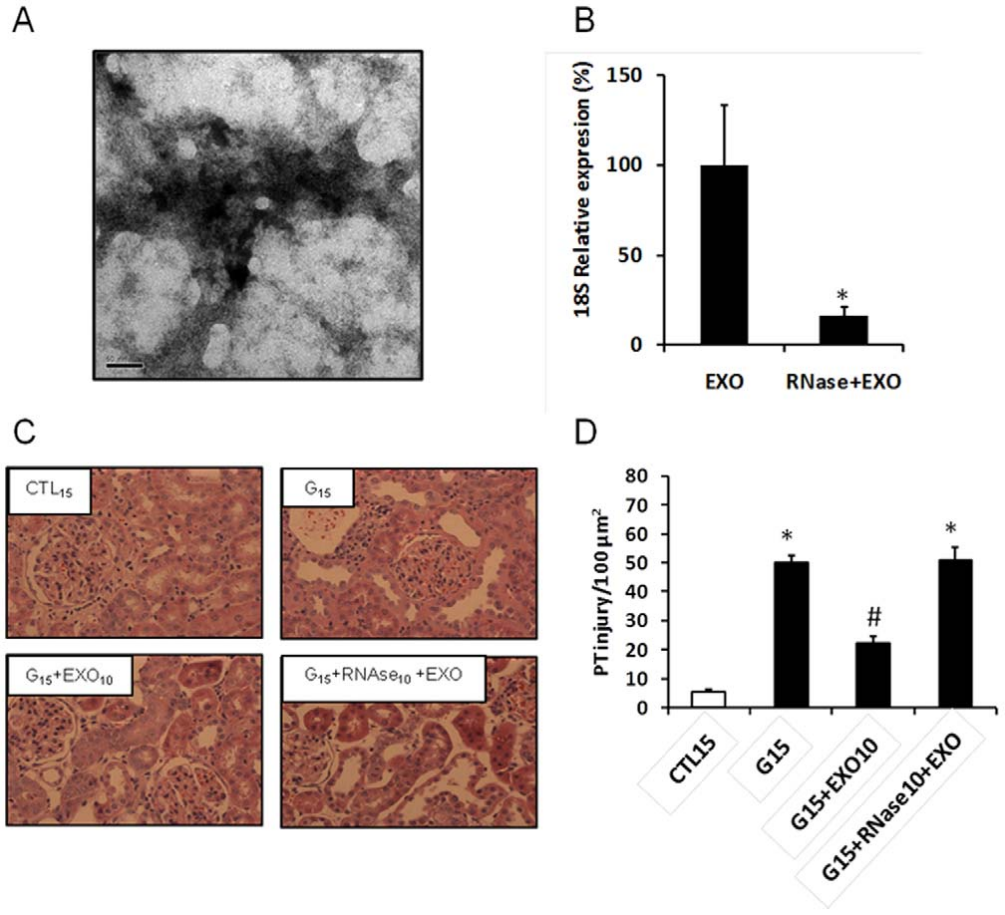
(A) Transmission Electron Microscopy of the exossomes-like microvesicles extracted from the BMSC conditioned medium. (B) RT-PCR for 18S RNA of exosome extracted from BMSC culture medium (EXO) untreated and treated with RNase (RNase+EXO). (C) The light micrographs of the kidney sections stained with hematoxylin eosin. (D) A graphic representation showing the quantitative analysis of the micrographs. The normal histology of the kidney tissue from control rats (CTL_15_) and rats treated with G for 15 days (G_15_). Some groups presented with massive tubular necrosis and unstained nuclei (G_15_, G_15_+RNAse+EXO_10_), while other groups presented with intensely stained nuclei (G_15_+EXO_10_). Data are expressed as the mean ± S.E.M. (* p<0.05) vs. CTL15d, (# p<0.05) vs. G15.

Figure 5A shows the transmission electron microscopy (TEM) of exosome-like microvesicles extracted from the conditioned medium. Figure 5B shows that the RNAse treatment significantly degraded the RNA carried by the exossome as shown by the housekeeping gene 18S expression. The treatment of the animals with these exosomes (100 µg/ml of protein) significantly induced cellular proliferation, as shown by Figure 5C and inhibited the tubular necrosis induced by G in these animals (Figure 5C and D). This effect was inhibited when the CM was treated with RNase and the exosomes were extracted (Figure 5C and D). This result suggests that the protective paracrine effect of the CM is mediated, at least in part, by exosome-like microvesicles released by the BMSC.

**Figure 6:**
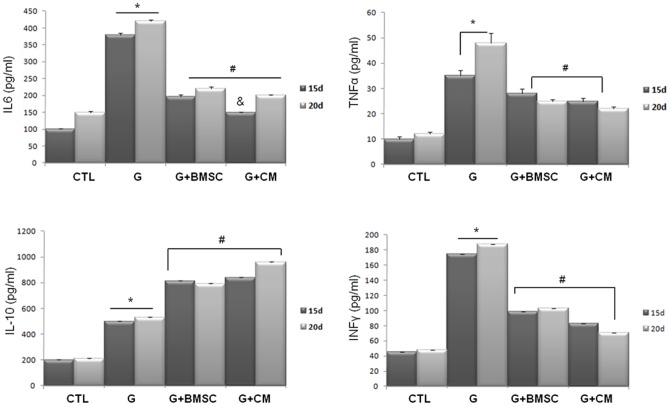
The plasma cytokine levels in the control (CTL15d and CTL20d), G-treated (G15 and G20) and G-treated rats that received BMSC (G15 or 20+BMSC10) or CM (G15 or 20+CM10). The data are expressed as the mean ± S.E.M. p<0.05 (*) vs. CTL15 or 20 d; (#) vs. G15 or 20 d; (&) vs. G+BMSC20d.

Several mechanisms have been proposed to explain the protective effect of BMSCs. There is evidence for both pro- and anti-inflammatory actions of BMSCs. To further address these mechanisms, we analyzed several pro- and anti-inflammatory cytokines in the serum of Wistar rats in the CTL, G, G+BMSC and G+CM groups after 15 and 20 days of treatment with the antibiotic to prove a sustained protective effect of the CM or the cells (Figure 6). There were significant increases in the pro-inflammatory cytokines IL6, INF-γ and TNF-α and a decrease in the anti-inflammatory cytokine IL10 in the G-treated groups, indicating the inflammatory effects of G. When the rats received BMSCs, the proinflammatory cytokines IL6, INF-γ and TNF-α decreased, while the anti-inflammatory cytokine increased compared to the G groups.

The same pattern was observed when the animals were treated with the CM. There was a decrease in IL6, INF-γ and TNF-α and an increase in IL10.

There were no significant differences in cytokine concentrations between the BMSC transplanted and CM-treated animals, suggesting that in our experimental model, the main effects of BMSCs are paracrine (Figure 6).

## Discussion

In clinical practice, an aminoglycoside injury is the most common cause for a nephrotoxic complication, and no significantly effective new therapy in humans has been introduced in decades [6].

In the current study, the animals were treated with G injections for up to 20 days to establish the time point of the G treatment that best reproduces what is observed in clinical practice. The treatment with G for 10 days was able to induce AKI [6] with an increase in serum creatinine, urea, FENa and apoptosis and a decrease in cell proliferation. Additionally, there was an increase in the pro-inflammatory and a decrease in the anti-inflammatory plasmatic cytokines that reflect the inflammatory process induced by G in the kidney. These results are in agreement with the findings of other studies [14]–[16].

To study the role of BMSCs on the treatment of G, BMSCs were administered on the 10^th^ day of treatment, and the rats continued receiving G for up to 20 days. During these periods, the cells were able to minimize the deleterious effects caused by G, thereby decreasing serum creatinine, urea, FENa and pro-inflammatory cytokines while increasing an anti-inflammatory cytokine. Additionally, there were decreases in the tubular lesions, as shown by the caspase 3 marker, along with increases in cell proliferation. Similar effects were also demonstrated by others, where the BMSCs were able to minimize renal damage in several models of AKI, such as the cisplatin, adriamycin and ischemia/reperfusion experimental models [17]–[19]. These authors reported that the BMSCs accelerate the structural recovery of the kidney after AKI, and, more importantly, that they confer therapeutic benefits.

We also evaluated whether BMSCs could prevent the AKI caused by G. It was observed that when BMSCs were administrated prior to treatment with G on the 5^th^ day of treatment, the full impact of G on the kidney was not blunted. Previous studies have shown that on the 5^th^ day of treatment with G, renal injury is already present [4], and BMSCs may be recruited to the injury site but not at a level sufficient to confer a protective effect.

The functional protection by BMSCs can be the consequence of the capacity of these cells to engraft the damaged kidney. We evaluated the presence of BMSCs in renal tissue by using the chromosome Y localization strategy. It was detected in all of the groups analyzed, indicating that BMSCs migrate to the local site of the injury and stay during the continuous injury period. However, the chromosome Y was not detected in the prevention group 24 hours before the insult with G, suggesting that the BMSCs were rapidly removed because no lesions were present in renal tissue to allow for the docking of stem cells. To our knowledge, this is the first report demonstrating that BMSCs are not able to prevent the injury induced by G prior to its use but that it is possible to reduce tissue injury if BMSC transplantation is performed during the early stages of AKI. However, because no improvement in the functional parameters was observed, it is necessary to further evaluate the impact of this effect on long-term nephrotoxicity. The paracrine effect of the BMSCs was confirmed by the administration of the CM on the 10^th^ day of G treatment, which resulted in reduced damage caused by the antibiotic in both functional and histopathological parameters. Strikingly, the CM was able to reduce the creatinine, urea, FE_Na_ and pro-inflammatory cytokine levels (e.g., IL6, IFT-γ and TNF-α) and to increase the anti-inflammatory cytokine IL10. These results suggest that BMSCs might be able to protect the injured tissue potentially by releasing factors with repair proprieties [20].

Other studies have demonstrated that micro- and mRNA and other factors are present in the microvesicles released by BMSCs and that these factors could mediate the paracrine effects [21]. In the present study, the CM was treated with RNase or trypsin; it was observed that the treatment of the CM with RNase, but not trypsin, completely abolished the protective effect, indicating the participation of RNA-like products in these processes as observed previously [21], [22]. Additionally, the same protective effect was observed when the animals were treated with these microvesicles. When exosome-like vesicles were extracted from the CM treated with RNase, the RNA carried in these exosomes was also degraded.

## Conclusions

Our results demonstrate that the effects of the BMSCs or their conditioned medium enhanced the recovery of renal function in an aminoglycoside AKI model and that the protective effects were mediated by RNA carried by the exosomes/microvesicles in the CM. These results provide a rationale for a potential therapeutic tool for nephrotoxic AKI treatment. Moreover, because the effect of the administration of the conditioned medium from BMSCs blunted the effects of G, it is possible to avoid cell transplantation in the treatment of this nephrotoxicity.

## Materials and Methods

### Culture of BMSCs

The experimental protocol of the present work was approved by the ethics committee of the Universidade Federal de São Paulo (20/2008). Bone marrow (BM) was obtained from 12-week-old male Wistar rats. Briefly, rats were euthanized, and the femurs and tibias were aseptically removed. The BM was flushed from the bone with DMEM medium (Sigma, St. Louis MO, USA) containing 5% FCS (Invitrogen, Paisley, Scotland) plus penicillin/streptomycin (100 U/ml to 0.1 mg/ml; Invitrogen) and then filtered through a 100-µm sterile filter (Falcon) to produce a single-cell suspension. The BMSCs were obtained by their tendency to adhere tightly to plastic culture dishes, as previously described [23], [24]. The BM cells were plated in culture dishes with DMEM plus 20% FCS and penicillin-streptomycin (100 U/ml to 0.1 mg/ml) and allowed to adhere for 24 h. Non-adherent cells were then removed. The medium was changed regularly every 3 days. After 2 to 3 weeks, adherent cells were detached by trypsin-EDTA (0.5 to 0.2 g/L; Invitrogen) and used for the *in vivo* experiments. Cells were utilized in the 4^th^ passage for all experiments. For the conditioned medium-treated groups, the BMSCs were cultured with DMEM without SBF for 24 hours. After this period, the CM was collected and administered (500 µl) into the caudal vein of the animals.

### Flow cytometry

The BMSCs (1–2×10^6^) then were characterized by an FACS analysis that included positive assays (1∶250) for anti-CD17-purified, anti-CD29-FITC, anti-CD44-FITC, anti-CD73- purified, anti-CD90-FITC, anti-CD106-PE, anti-STRO1-purified and negative assays for anti-CD11b-purified, anti-CD45-purified and anti-CD34-FITC (all purchased from BD Pharmagen, California, USA); one negative control tube with a cell suspension was used as a control. The cells were incubated with a purified antibody, washed twice with PBS buffer and incubated with streptavidin-APC (Becton Dickinson Company, NJ, USA) for 20 minutes. After the incubation, the cells were washed again with PBS buffer and resuspended with 500 µl of PBS for the FACS analysis. The flow cytometry experiments were performed in duplicate.

### In Vitro Differentiation Assays

The BMSCs were studied to verify their mesenchymal potential to differentiate into osteoblasts and adipocytes. The BMSCs were grown until confluence, and the medium was replaced with the inductive medium consisting of Iscove's modified Dulbecco's medium, 20% FCS, 100 U/ml penicillin and 100 µg/ml streptomycin supplemented with specific differentiation reagents as follows:

#### Osteogenesis

The cells were incubated for 3 weeks, and the culture media, consisting of 10 mM ß-glycero-phosphate, 50 µg/ml ascorbic acid 2-phosphate, and 10^−9^ M dexamethasone (all from Sigma, St. Louis MO, USA), was changed twice a week. The cells were fixed with 10% formalin, and the mineralization (presence of calcium-rich hydroxyapatite) of the extracellular matrix was assessed by staining for 20 min with 2% wt/vol Alizarin Red S, adjusted to a pH of 4.1 with ammonium hydroxide (all reagents were from Sigma) [24], [25].

#### Adipogenesis

Cells were incubated for 3 weeks with 5 µg/ml insulin (Sigma) and 10^−9^ M dexamethasone (Sigma). The adipogenic differentiation was visualized in phase-contrast microscopy by the presence of highly refractive intracellular lipid vacuoles. Oil Red O (Sigma) staining was used to assay the accumulation of lipid droplets in these vacuoles [26].

### Groups

#### BMSCs Protocol

Female rats weighing 230–250 g (N = 10 for each group) were divided into groups as shown in Figure 2. The control group (CTL) was treated with daily *i.p.* injections of a vehicle (water), while the G-groups received gentamicin (40 mg/Kg BW) continuously for 10, 11, 12, 15 or 20 days. For the prevention groups, the rats received BMSC 1×10^6^ *i.v.* injections 24 hours before (G_10d_+BMSC_−1_) or on the 5^th^ day (G_10d_+BMSC_5_) of a 10-day treatment with G. For the treated groups, the female Wistar rats received 1×10^6^ BMSC *i.v.* injections on the 10^th^ day of G treatment and continued to receive G for an additional 1 (G_11_+BMSC_10_), 2 (G_12_+BMSC_10_), 5 (G_15_+BMSC_10_) or 10 days (G_20_+BMSC_10_) because it is known that the rats may recover spontaneously from AKI induced by G. The BMSC concentration was chosen according to previous protocols [27], [28]. At the end of these treatments, the rats were placed in individual metabolic cages for 24 hours to collect the blood and urine samples used for the creatinine, urea and sodium measurements.

### Conditioned Medium Protocol

As shown in Figure 1, the female rats received G (40 mg/Kg/BW, *i.p.*, daily) or water (CTL) for 15 or 20 days (N = 10/group). On the 10^th^ day of the G treatment, the animals received 500 µl of a single dose of CM delivered intravenously (BMSCs cultured for 12 h with DMEM). In some experiments, the BMSCs were cultured for 12 h with trypsin at a concentration of 100 ug/ml (CM+TPS) or RNase A at a concentration of 40 ug/ml, 280 Units (CM+RNAse) in DMEM without FBS. After this period, the medium was collected, centrifuged and filtered to remove the cells, treated with an RNase inhibitor (500 U) and administered to the rats. Additionally, the rats were treated with DMEM containing only RNase and trypsin in the same concentrations to account for the effect of the treatments.

Exosomes were isolated as described [29]. Briefly, the culture medium collected from the BMSCs was centrifuged for 5 min at 800×g and 10 min at 2000×g. The resultant supernatants were filtered with 0.1 µm pore filters (Millipore), which collected only 100 nm vesicles or smaller, followed by an ultra-centrifugation at 100,000×g for 60 min in a Beckman l-90 ultracentrifuge (Beckman Coulter). The resulting pellets were suspended in PBS, pooled and again ultracentrifuged at 100,000×g for 60 min. The final pellets of exosomes were suspended in 500 µl of Trizol for RNA extraction, in RIPA buffer for protein quantification or in PSB for animal's treatment [30]. The exosomes quantification was analyzed by protein concentration and the animals were treated with 100 µg/ml of protein.

The protein quantification was analyzed by BCA kit (Bioagency, USA) according to Lowry methodology. The experiments were conducted according to the manufacturer's instructions.

### Biochemical and histological analyses

Blood and 24-h urine samples were collected for the analysis of creatinine (Cr), urea (U) and FE_Na_ dosages. Cytokines (IL10, TNFα and INTγ) were analyzed in the group of animals that received 15 days of treatment with G. The kidneys were examined by HE, KI67 and caspase 3 staining.

Creatinine was determined by a colorimetric method based on the Jaffé reaction [31] using creatinine assay kits (Labtest, Minas Gerais, Brazil).

Urea was determined using Urease-Labtest [32], and the renal tubular function was evaluated by measuring the fractional excretion of sodium (FE_Na_). The sodium concentrations in the urine and plasma were determined with a Klina flame photometer (Becton Dickinson Company, NJ, USA).

ELISA kits (R&D Systems, Minneapolis, Minnesota, USA) were used to determine the concentrations of cytokines (TNF-α, INF-γ, IL6 and IL10). The plasma levels of cytokines in 10 animals were determined, as the production of cytokines by epithelial or inflammatory cells can be detected in the blood stream. The experiments were conducted according to the manufacturer's instructions.

After the experimental protocol, the animals were anesthetized for renal perfusion through the aorta, and the kidneys were removed for histological, immunofluorescence and immunochemistry studies.

The kidneys were bisected along the non-hilar axis and fixed in 10% phosphate-buffered formalin (Invitrogen, São Paulo, Brazil). These tissues were subsequently embedded in paraffin (Casas Americanas, São Paulo, Brazil), sectioned, and stained with hematoxylin and eosin (Merck, New Jersey, USA). The kidneys were examined microscopically by an independent pathologist and one of the investigators.

Histopathological data were analyzed for tubular necrosis, proteinaceous casts, and medullar congestion, as suggested by Solez *et al* [33], and the changes were quantified.

### Immunofluorescence microscopy

To verify the presence of the BMSCs extracted from the male rats and to characterize their phenotype in the kidney of the female recipients (Y chromosome), sections of the glomeruli, proximal tubular brush border, and renal interstitial area were stained and analyzed.

The kidneys were fixed in Dubosq-Brazil fluid, routinely processed, and paraffin wax embedded. The slides were dewaxed by xylene, taken through graded alcohol washes (at 100, 90, 70, and 60%) to PBS, and incubated with trypsin (0.1% in CaCl_2_ 0.1%) at 37°C for 15 min.

Frozen sections were treated with 2% bovine serum albumin and incubated with goat anti-mouse IgG antibody (Invitrogen). Then, the sections were stained with the primary rat polyclonal anti-Y-chromosome IgG antibody (1∶200) (Santa Cruz Biotechnology, New Jersey, USA) overnight at 20°C. A secondary FITC-conjugated rabbit anti-mouse IgG antibody (1∶2000; Invitrogen) was added before counterstaining with DAPI, and slides were mounted with Dako Cytomation fluorescent mounting medium (Dako Cytomation, CA, USA).

Immunofluorescence staining was visualized and recorded with an Olympus Floview 1000 confocal microscope (Olympus® Bx50, Olympus, Tokyo, Japan).

In additional experiments, the tubular basement membrane structures were stained for a more precise localization of Y chromosome–positive cells.

Proliferation and apoptosis were identified by using immunohistochemistry staining. The sections were boiled twice for 5 min in citrate buffer and were blocked for 30 min in 1% BSA (Sigma, St. Louis MO, USA) to reduce the background. The tissue sections were processed for free-floating immunohistochemistry according to the streptavidin-biotin-peroxidase method.

Briefly, the sections were incubated in 1% H_2_O_2_ for 30 min to quench endogenous peroxidase activity, followed by a permeabilization with a Tris–Triton 0.1% solution, and then were incubated in 1% BSA for 30 min to block any unspecific binding. The sections were then incubated overnight at 4°C with primary antibodies against anti-KI67 (rat monoclonal, 1∶200; from Dako) or cleaved anti-caspase 3 (rat monoclonal, 1∶200; Dako) diluted in Tris HCl 0.1 M (pH 7.4) containing 0.1% bovine serum albumin (BSA) and 0.3% Triton X-100. In the control experiments, the first antibody was omitted or replaced by normal serum.

After rinsing, the tissues were incubated for 10 min at room temperature with a secondary antibody using a DAKO LSAB™ Kit (Dako Cytomation, CA, USA), a labeled streptavidin-biotin reagent system that includes a biotinylated secondary antibody plus horseradish-labeled streptavidin-biotin reagents. After rinsing with Tris buffer, the complex was visualized with a DAKO (Dako Cytomation, CA, USA) liquid 3,3′-diaminobenzidine chromogen solution.

The tissues were transferred to a buffer solution to stop the reaction, rinsed again in distilled water and mounted onto silanized glass slides. The sections were dehydrated through graded alcohol washes, cleared in xylene and covered with a cover slip.

All labeled (staining light to dark brown) tubular cells of each section were counted and averaged for total immunoreactive cells. Three to four sections per structure or tubular along the kidney cortex were counted and averaged for each rat. The values obtained are expressed as the number of positively stained tubulars ± SEM for 5 animals per group.

Digital photomicrographs were taken through a Nikon Eclipse E600 upright microscope equipped with Plan Apo objectives and connected to a Dell workstation computer through the PixeLINK® Microscope Camera Software (Ottawa, Canada).

### RNA isolation, Reverse transcription and Quantitative Real Time PCR

Total RNA from the BMSC culture medium treated with RNase (40 µg/ml) was extracted using a Trizol technique (Invitrogen Life Technologies) according to the manufacturer's instructions and published protocol [32]. Reverse transcription was performed using a High Capacity cDNA Reverse Transcription kit for real-time PCR (Applied Biosystems). Primers for 18S (forward, 5′- GAACCAGAGCGAAAGCATTTGCCA -3′; reverse, 5′- TCGGCATCGTTTATGGTCGGAACT -3′) were designed and synthesized by Prodimol. A real-time analysis was performed on an ABI Prism 7900 (Applied Biosystems) using the SYBR Green PCR Master Mix for real-time PCR (Applied Biosystems). The relative expression was determined by a standard curve.

### Transmission Electron Microscopy (TEM)

Exosome pellets were prepared for negative staining. Exosome pellet (3 µl) was gently placed on 200-mesh Formvar-coated copper grids, allowed to adsorb for 4–5 min, and processed for standard uranyl acetate staining. The grid was washed with three changes of PBS and allowed to semidry at room temperature before observation in TEM (Jeol 1200 EXII).

### Viability

The BMSC viability was analyzed by a Trypan Blue extrusion followed by a counting of the cells. The results are expressed as percentages.

### Statistical analysis

The results (unless otherwise noted) are expressed as the mean ± standard error of the mean (SEM). For the renal functional studies, One-Way variance analysis (ANOVA) was used and was followed by Dunnett's test for multiple comparisons. The histology and immunohistochemistry data were analyzed using the nonparametric Kruskal-Wallis test or Mann-Whitney test, as appropriate. The statistical significance level was defined as *P*<0.05.

The statistical analyses of the flow cytometry were performed in the (Cell Quest program, BD Bioscience USA), and these results are presented as cell percentages. An ANOVA was conducted to test the comparison among groups using Bartlett's test for equal variances and Tukey's multiple comparison tests.

## Supporting Information

Figure S1 Light micrographs of kidney sections stained with immunofluorescence for Y chromosome (100×). (CTL10) Female Wistar rats treated with G vehicle during 10 days. (G10) Female Wistar rats treated with G during 10 days. (G11,12 or20+BMSC10) Female Wistar rats treated with G during 11, 12 or 20 days and BMSC in 10th day. The arrows showed the presence of Y chromosome in the epithelial tubule cells. Images magnified 100×.
(TIF)

Figure S2 Numbers of proximal tubules injury for 100 µ2 in female rats treated with G (40 mg/Kg BW) during 10, 12, 15 or 20 days and the 10th day, treated with BMSC (1×106, iv). *p<0.05 vs. G10,12,15 or 20 d.
(TIF)
