# Supplementary figures and images for: Bone Marrow-Derived Mesenchymal Stem Cells Repaired but Did Not Prevent Gentamicin-Induced Acute Kidney Injury through Paracrine Effects in Rats
Source: PLoS One. 2012 Sep 6;7(9):e44092. doi: 10.1371/journal.pone.0044092 (PMC3435420; doi:10.1371/journal.pone.0044092)

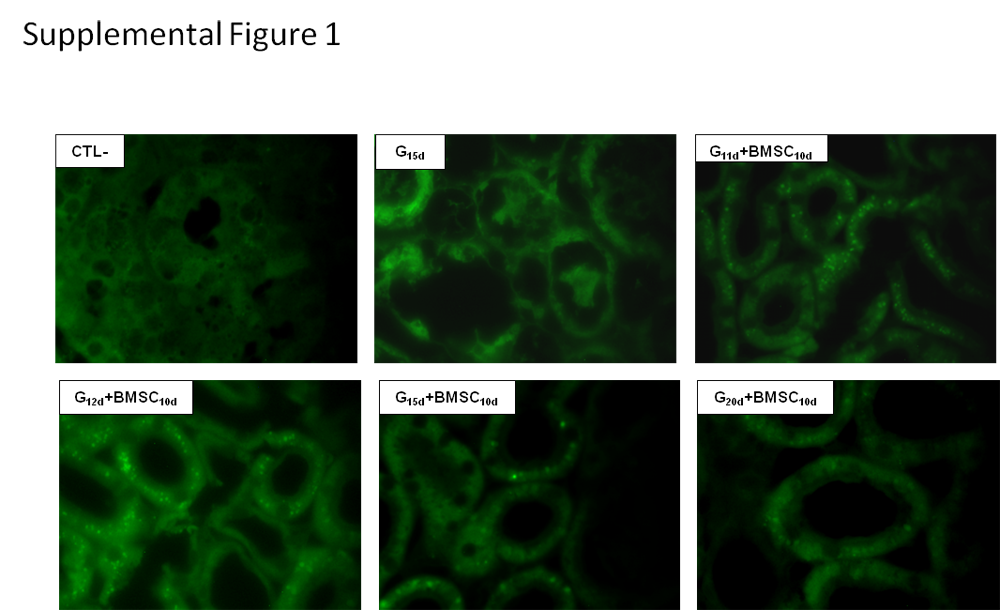

Supplement: Figure S1 — Light micrographs of kidney sections stained with immunofluorescence for Y chromosome (100×). (CTL10) Female Wistar rats treated with G vehicle during 10 days. (G10) Female Wistar rats treated with G during 10 days. (G11,12 or20+BMSC10) Female Wistar rats treated with G during 11, 12 or 20 days and BMSC in 10th day. The arrows showed the presence of Y chromosome in the epithelial tubule cells. Images magnified 100×. (TIF) [file pone.0044092.s001.tif]

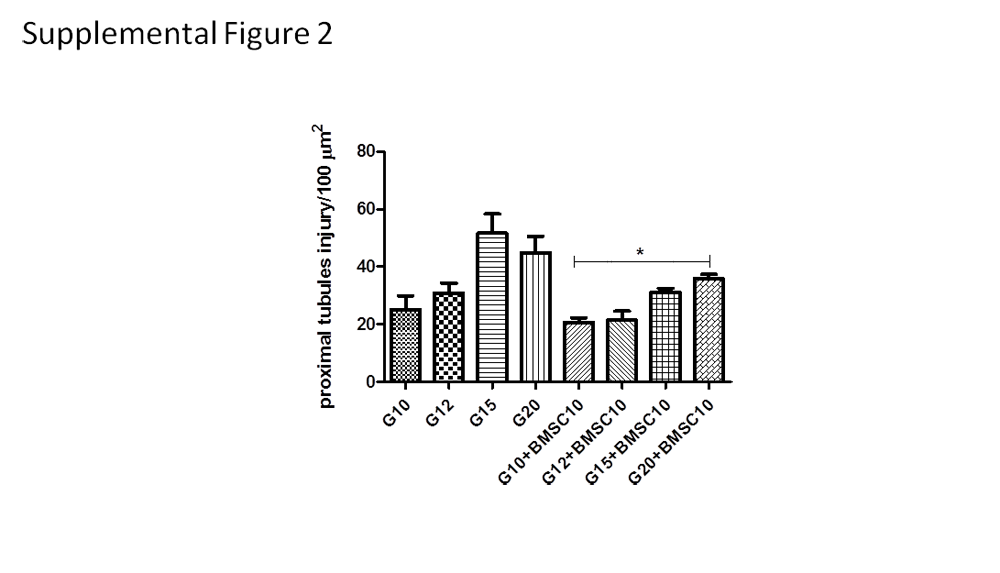

Supplement: Figure S2 — Numbers of proximal tubules injury for 100 µ2 in female rats treated with G (40 mg/Kg BW) during 10, 12, 15 or 20 days and the 10th day, treated with BMSC (1×10^6^, iv). *p<0.05 vs. G10,12,15 or 20 d. (TIF) [file pone.0044092.s002.tif]
